# Tributyltin Oxide Exposure During *in vitro* Maturation Disrupts Oocyte Maturation and Subsequent Embryonic Developmental Competence in Pigs

**DOI:** 10.3389/fcell.2021.683448

**Published:** 2021-06-28

**Authors:** Yue Xiao, Bao Yuan, Weiyi Hu, Jiajia Qi, Hao Jiang, Boxing Sun, Jiabao Zhang, Shuang Liang

**Affiliations:** Department of Animal Sciences, College of Animal Sciences, Jilin University, Changchun, China

**Keywords:** tributyltin oxide, oxidative stress, porcine oocyte, quality, impairments

## Abstract

Tributyltin oxide (TBTO), an organotin compound, has been demonstrated to have toxic effects on several cell types. Previous research has shown that TBTO impairs mouse denuded oocyte maturation. However, limited information is available on the effects of TBTO exposure on livestock reproductive systems, especially on porcine oocytes in the presence of dense cumulus cells. In the present research, we evaluated the effects of TBTO exposure on porcine oocyte maturation and the possible underlying mechanisms. Porcine cumulus-oocyte complexes were cultured in maturation medium with or without TBTO for 42 h. We found that TBTO exposure during oocyte maturation prevented polar body extrusion, inhibited cumulus expansion and impaired subsequent blastocyst formation after parthenogenetic activation. Further analysis revealed that TBTO exposure not only induced intracellular reactive oxygen species (ROS) accumulation but also caused a loss of mitochondrial membrane potential and reduced intracellular ATP generation. In addition, TBTO exposure impaired porcine oocyte quality by disrupting cellular iron homeostasis. Taken together, these results demonstrate that TBTO exposure impairs the porcine oocyte maturation process by inducing intracellular ROS accumulation, causing mitochondrial dysfunction, and disrupting cellular iron homeostasis, thus decreasing the quality and impairing the subsequent embryonic developmental competence of porcine oocytes.

## Introduction

Increasing amounts of evidence indicate that environmental pollutants may harm human health because these contaminants can gradually accumulate in the human body after consumption of tainted water and food (Carbery et al., 2018; Kuckelkorn et al., 2018). In recent years, the organotin compound tributyltin (TBT), a bioaccumulative and persistent environmental pollutant, has become a subject of great concern (Pereira et al., 2019; Jie et al., 2021). TBT is an active ingredient of marine antifouling paints and is broadly applied to boats and ships. Humans can be exposed to TBT through consumption of contaminated fish and seafood, and the ingested TBT can affect the reproductive system (Lang Podratz et al., 2012; Xiao et al., 2018) and immune system (Frouin et al., 2008; Brown and Whalen, 2015). Previous studies have reported that TBT exposure may disrupt oogenesis and serotonin synthesis (Xiao et al., 2018), decrease ovarian weight, unbalance the levels of female sex hormones, and elevate the numbers of atretic follicles and corpora lutea (Lang Podratz et al., 2012). Furthermore, TBT exposure inhibits estrogen receptor-dependent transcriptional activation and prevents the interaction between the human estrogen receptor β ligand-binding domain (LBD) and steroid receptor coactivator-1 in yeast (Cho et al., 2012).

TBT oxide (TBTO) is a TBT compound that has been widely used as an antifouling agent in marine paints, an agent in wood preservation and a stabilizer in the plastic industry (Osman and van Loveren, 2012). TBTO exposure may impair spermatogenesis in marine fish (Mochida et al., 2007), induce immunotoxicity, suppress total ATPase activity in mice and rats (Elsabbagh et al., 2002; Baken et al., 2006), and cause thymus atrophy in rodents (van Kol et al., 2012). TBTO exposure induces oxidative stress, apoptosis, and endoplasmic reticulum (ER) stress in the human T lymphocyte cell line CTLL-2 and immature rat thymocytes (Raffray and Cohen, 1991; Katika et al., 2011; Schmeits et al., 2013). Previous studies have shown that TBTO exposure also has a toxic effect on the male reproductive system: It can cause severe histological damage to the testes, including malformation of somatic cells around the seminal duct, and reduce the numbers of spermatids and spermatozoa (Mochida et al., 2007). As TBTO is a model immune poison, TBTO exposure can inhibit chemotaxis associated with the chemokine CXCL12, thereby affecting the migration of white blood cells (Shao et al., 2016). In addition, TBTO exposure can reduce the immunotoxicity of interleukin 2 by blocking the mTOR pathway (Osman and van Loveren, 2014). Research has shown that TBTO exposure affects proliferation and energy sensor pathways by downregulating MAPK, matrin-3 and the ribonucleotide reductase subunit RRM2, which are implicated in cell proliferation in mouse thymoma cells (Osman and van Loveren, 2012). An immunocytological analysis in Jurkat cells has shown that TBTO exposure induces the expression of the key oxidative stress response genes NRF2 and KEAP1 (Katika et al., 2011). Previous *in vitro* experiments have shown that TBTO exposure can increase the occurrence of abnormal spindle organization and chromosome misalignment and induce mitochondrial dysfunction, oxidative stress, and apoptosis, thus impairing oocyte quality in mice (Yang et al., 2019).

Oocyte maturation is a complex process, and the quality of mature oocytes plays a decisive role in preimplantation embryonic development (Sen and Caiazza, 2013; Keefe et al., 2015). However, during this process, oocytes are sensitive to exogenous toxic agents, which can disrupt oocyte maturation and interfere with preimplantation embryonic developmental competence. Compared with rodents, pigs are more similar to humans both physiologically and metabolically, and it is more practical to use porcine oocytes than to use rodent oocytes as experimental objects for reproductive toxicology analyses (Magnusson, 2005; Santos et al., 2014). Elucidation of the effects of TBTO on porcine germ cell development will provide useful information for human biomedical research. In the present research, porcine oocytes were used as a model to analyze the effects of TBTO on mammalian oocyte quality. We hypothesized that TBTO exposure during porcine oocyte maturation would decrease oocyte quality and subsequent embryonic developmental competence.

## Materials and Methods

All drugs and reagents used in the present research were obtained from Sigma-Aldrich (St. Louis, MO, United States) unless otherwise stated.

### Porcine Oocyte Collection and *in vitro* Maturation (IVM)

Porcine ovaries were obtained from a local slaughterhouse and transported to the laboratory in sterile 0.9% saline at 30–35°C. Cumulus-oocyte complexes (COCs) were obtained by aspirating 3∼8 mm antral follicles with a syringe. COCs with three or more layers of uniformly distributed cumulus cells were collected using Tyrode’s lactate-hydroxyethyl piperazine ethane sulfonic acid (TL-HEPES) medium supplemented with 0.1% polyvinyl alcohol (PVA, w/v) and 0.05 g/L gentamycin under a stereomicroscope (S22-LGB, Nikon). The IVM medium consisted of tissue culture medium 199 (TCM-199, Invitrogen, Carlsbad, CA, United States) supplemented with 10% (v/v) porcine follicular fluid, 10 IU/mL follicle-stimulating hormone (Ningbo No. 2 Hormone Factory, China), 10 IU/mL luteinizing hormone (Ningbo No. 2 Hormone Factory, China), 0.91 mM Na pyruvate, 10 ng/mL EGF, and 75 mg/mL kanamycin. The IVM medium was overlain with mineral oil, and the oocytes were cultured in an incubator containing 5% CO_2_ at 100% humidity and 38.5°C for 42 h. For TBTO exposure, working solutions of 5, 25, and 50 μM TBTO were added to the medium during porcine oocyte IVM.

At the end of IVM, the extent of cumulus cell expansion was photographed by a microscope (IX73, Olympus, Tokyo, Japan). The extent of cumulus expansion was assessed by measuring the oocyte areas with cumulus cells using NIH ImageJ software (National Institutes of Health, Bethesda, MD, United States). Then, the COCs with an expanded cumulus corona cell complex were removed with 0.1% hyaluronidase. Polar body extrusion (PBE) of oocytes was examined under a stereomicroscope (S22-LGB, Nikon).

### Assessment of Cumulus Cell Apoptosis

For analysis of apoptosis, porcine cumulus cells were removed from COCs with expanded cumulus cells using 0.1% hyaluronidase and collected using a 1.5 mL centrifuge tube at the end of the IVM period. Apoptosis in the porcine cumulus cells in each group was detected by flow cytometry using a FITC Annexin V Apoptosis Detection Kit (556547, BD Biosciences) and analyzed by flow cytometry (BD Biosciences) within 1 h.

### Western Blotting Analysis

Total protein was extracted from porcine cumulus cells using RIPA lysis buffer (AR0102, Boster) with a broad-spectrum protease inhibitor mixture (AR1182, Boster) according to the manufacturer’s instructions. Then, the protein concentration of each group was measured by a BCA Protein Assay Kit (S7705, TIANGEN) according to the manufacturer’s instructions, and total protein was separated by sodium dodecyl sulfate polyacrylamide gel electrophoresis (SDS-PAGE) and transferred to a polyvinylidene difluoride (PVDF) membrane. Blocking buffer (WLA066a, Wanleibio) was used to block the transferred membranes, and the membranes were incubated overnight with primary antibodies against GAPDH (CST, #2118S), TUBULIN (Proteintech, 10094-1-AP), BCL-2 (Wanleibio, WL01556), BAX (Wanleibio, WL01637), COX2 (Proteintech, 12375-1-AP) and HAS2 (Bioss, bs-11290R). After washing three times with Tris-buffered saline with Tween 20 (TBST), the PVDF membranes were incubated with secondary antibodies. The immunoblots were developed using SuperSignal^TM^ West Pico PLUS Chemiluminescent Substrate (34580, Thermo), and the signal intensities were captured by a Tanon 5200 chemiluminescence/fluorescence image analysis system. Protein levels were quantified using ImageJ software.

### Parthenogenetic Activation (PA) and *in vitro* Culture (IVC)

Porcine oocyte PA was induced according to our previously described procedures (Qi et al., 2020). Briefly, denuded oocytes with polar bodies were subjected to electrical activation at 110 V for 60 μs twice with a 0.1 s interval. After that, the oocytes were transferred to IVC medium (bicarbonate-buffered porcine zygote medium (PZM)-5 comprising 4 mg/mL BSA) supplemented with 7.5 μg/mL cytochalasin B and cultured for 3 h to suppress extrusion of the pseudosecond polar body. Next, the oocytes were thoroughly washed and cultured in four-well plates in IVC medium covered with mineral oil for 7 days at 38.5°C under 100% humidity and an atmosphere of 5% CO_2_ without changing the medium. Cleavage and blastocyst formation rates were analyzed under a stereomicroscope at 2 days, 6 days, and 7 days.

### Assessment of Blastocyst Diameters and Total Cell Numbers

On day 6, the blastocysts were photographed with a microscope (IX73, Olympus, Tokyo, Japan), and the blastocyst diameters in each group were analyzed with NIH ImageJ software (National Institutes of Health, Bethesda, MD, United States). To determine the total cell numbers in blastocysts, day-7 blastocysts derived from parthenogenetically activated embryos were collected and fixed in 3.7% paraformaldehyde in PBS-PVA medium for 30 min. Then, the blastocysts were stained with 10 μg/mL Hoechst 33342 to label the nuclei. After that, the stained blastocysts were gently mounted onto glass slides, examined, and photographed with a microscope under fluorescent light. The total cell numbers in blastocysts were analyzed with NIH ImageJ software.

### Assessment of Oocyte Intracellular ROS Levels and Mitochondrial Membrane Potential (MitoMP)

At the end of the IVM period, intracellular ROS levels and MitoMP were measured with an ROS detection kit (Thermo Fisher Scientific, C400) and a JC-1 MitoMP detection kit (Dojindo, MT09) following the instructions of the kits. Images of the fluorescence signals were captured as TIFF files using a digital camera connected to a fluorescence microscope. The fluorescence signal intensities of the oocytes in each group were analyzed with NIH ImageJ software.

### Measurement of Oocyte Intracellular ATP Levels

Intracellular ATP levels were measured using an ATP Detection Kit (Beyotime, S0027). Briefly, porcine oocytes were collected from each group and lysed with 200 μL of lysis buffer at the end of the IVM period. Next, the cell lysates were centrifuged at 12,000 rpm at 4°C for 5 min, and the supernatant was taken for subsequent analysis. Then, 100 μL of ATP working solution and 20 μL of supernatant were added to 96-well opaque plates, and the well contents were analyzed with a luminometer (Tecan, Infinite M200 Pro).

### Assessment of Oocyte Intracellular Ferrous Ion Levels

Intracellular ferrous ion levels were examined at the end of the IVM period. The oocytes in each group were thoroughly washed in prewarmed PBS-PVA medium and assessed using the fluorescent probe Ferro Orange (Dojindo, F374) for 30 min. Images of the fluorescence signals were captured as TIFF files using a digital camera connected to a fluorescence microscope. The fluorescence signal intensities of the oocytes in each group were analyzed with NIH ImageJ software.

### Statistical Analysis

Statistical analysis was carried out by SPSS 19.0 software. All experiments were repeated at least three times. The data were analyzed using Student’s *t*-test or ANOVA and are presented as the mean ± standard deviation (SD). *p*-values < 0.05 were considered statistically significant.

## Results

### TBTO Exposure During the IVM Period Impairs Porcine Oocyte Maturation and Cumulus Expansion and Increases Apoptotic Cumulus Cell Proportion

**FIGURE 1:**
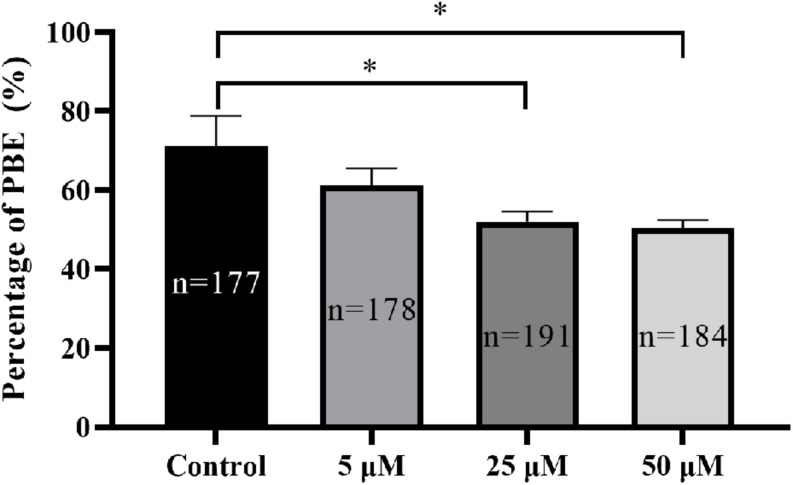
Effects of TBTO exposure on porcine oocyte maturation. Percentage of oocytes with PBE in each group. The numbers of oocytes examined from the different groups are indicated by the bars. ^∗^*p* < 0.05 compared to the control.

To determine the potential effect of TBTO exposure on porcine oocyte maturation during the IVM period, COCs were treated with increasing concentrations of TBTO (5, 25, and 50 μM), and the percentages of oocytes with PBE were analyzed at the end of IVM. The results revealed that TBTO exposure dose-dependently decreased the percentage of oocytes with PBE (control: 71.08% ± 7.70%, 5 μM: 61.17% ± 4.37%, 25 μM: 52.06% ± 2.54%, 50 μM: 50.48% ± 1.99%; *p* < 0.05) (Figure 1).

**FIGURE 2:**
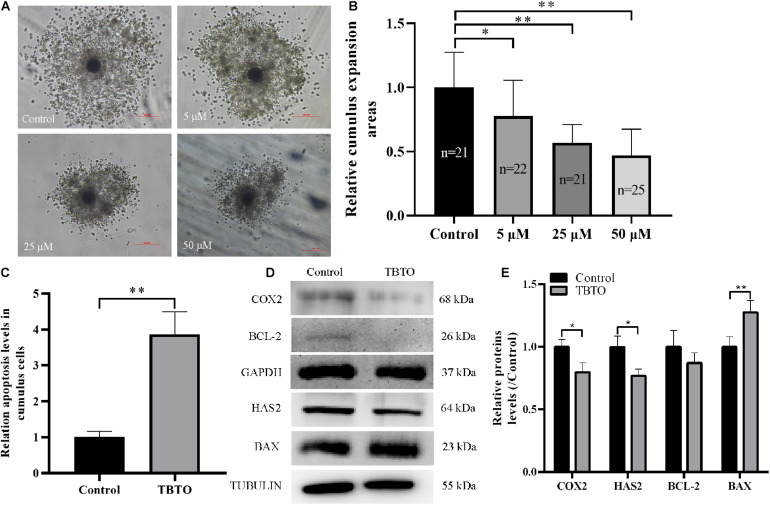
Effects of TBTO exposure on the cumulus expansion status of porcine oocytes. **(A)** Representative images of cumulus expansion status in the control and TBTO exposure groups at the end of the IVM period. Scale bar = 200 μm. **(B)** Relative cumulus expansion areas in the control and TBTO exposure groups. **(C)** Relative apoptosis levels in cumulus cells between control and TBTO exposure group by flow cytometry analysis. **(D)** Western blotting was used to detect the protein levels in cumulus cells between control and TBTO exposure group. **(E)** Histogram showing the relative levels of several proteins in TBTO exposure group compared to the reference proteins in the control group. The numbers of COCs examined from the different groups are indicated by the bars. ^∗^*p* < 0.05; ^∗∗^*p* < 0.01 compared to the control.

Cumulus expansion is necessary for oocyte maturation and is often used as an indicator of oocyte quality at the end of IVM. Therefore, we analyzed whether TBTO exposure affected cumulus expansion during porcine oocyte maturation. Representative images of cumulus expansion status are shown in Figure 2A. The results obtained at the end of IVM showed that TBTO exposure decreased the relative cumulus expansion area of COCs in a dose-dependent manner (*p* < 0.05, Figure 2B). Flow cytometry analysis revealed that 25 μM TBTO exposure increased the relative proportion of apoptotic cumulus cells in porcine COCs (*p* < 0.05, Figure 2C). Furthermore, western blotting analysis showed that the levels of the cumulus expansion-related factors COX2 and HAS2 was downregulated, and the levels of the apoptosis-related factors BAX in cumulus cells exposed to 25 μM TBTO was compared with that in cumulus cells in the control group (Figures 2D,E). These results suggest that TBTO exposure has a direct negative effect on porcine oocyte maturation in a dose-dependent manner. Given the findings, 25 μM TBTO was selected for use in all subsequent experiments.

### TBTO Exposure During the IVM Period Impairs Subsequent *in vitro* Embryo Development of Porcine Oocytes After PA

**FIGURE 3:**
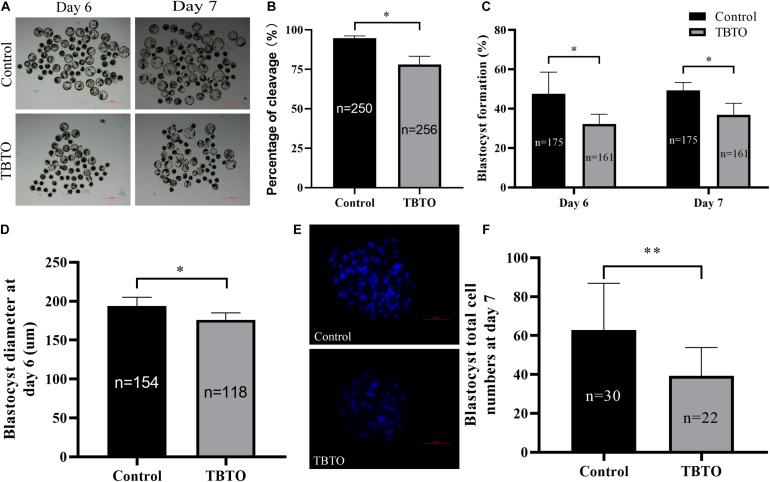
Developmental competence of porcine oocytes after TBTO exposure. **(A)** Representative images of PA embryo development on day 6 (top) and day 7 (bottom) in the control and TBTO exposure group at the end of the IVM period. Scale bar = 500 μm. Percentages of cleavage **(B)** and blastocyst formation **(C)** in the control and TBTO exposure group. **(D)** Blastocyst diameter in the control and TBTO exposure group. **(E)** Representative images of Hoechst 33342 staining of blastocysts on day 7 in the control and TBTO exposure group. Scale bar = 100 μm. **(F)** Blastocyst total cell numbers in the control and TBTO exposure group. The numbers of embryos examined from the different groups are indicated by the bars. ^∗^*p* < 0.05; ^∗∗^*p* < 0.01 compared to the control.

We further assessed whether TBTO exposure during the IVM period impaired the developmental competence of parthenogenetically activated porcine embryos. The results revealed that TBTO exposure had a negative effect on the developmental competence of the embryos (Figure 3A). As shown in Figures 3B,C, the cleavage rate (93.77% ± 0.74% vs. 77.98% ± 5.24 on day 2; *p* < 0.05) and blastocyst formation rate (50.71% ± 11.08% vs. 32.15% ± 5.01% on day 6 and 49.38% ± 3.90% vs. 36.85% ± 5.94% on day 7; *p* < 0.05) of the parthenogenetically activated embryos generated from matured oocytes were significantly lower for the TBTO-exposed group than for the control group. The diameter (Figure 3D, 193.98 ± 11.05 μm vs. 176.21 ± 8.93 μm on day 6; *p* < 0.05) and total cell numbers (Figures 3E,F, 63.68 ± 24.03 vs. 39.27 ± 14.64; *p < 0.05*) of blastocysts derived from parthenogenetically activated embryos were significantly lower in the TBTO-exposed group than in the control group.

### TBTO Exposure During the IVM Period Induces Oxidative Stress in Porcine Oocytes

**FIGURE 4:**
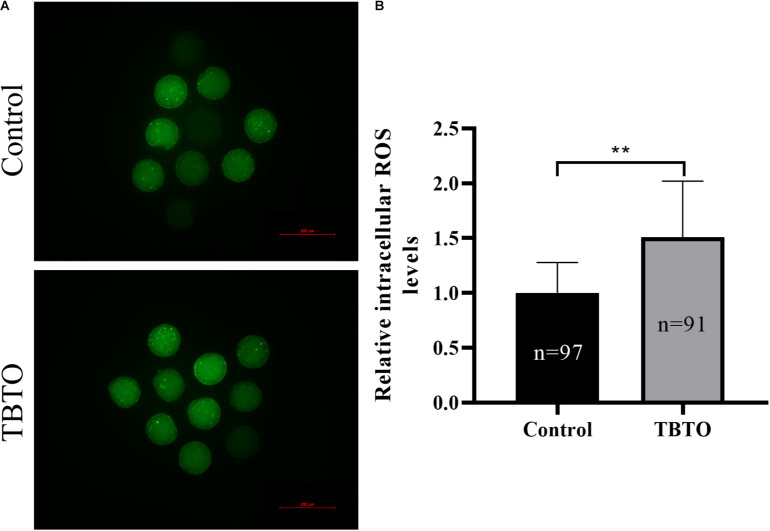
Effects of TBTO exposure on intracellular ROS generation in porcine oocytes. **(A)** Representative fluorescence images showing intracellular ROS levels in porcine oocytes in the control and TBTO exposure group at the end of the IVM period. Scale bar = 200 μm. **(B)** Quantification of relative intracellular ROS levels in porcine oocytes from the control and TBTO exposure group. The numbers of oocytes examined from the different groups are indicated by the bars. ^∗∗^*p* < 0.01 compared to the control.

Intracellular ROS levels were measured by assessing DCFH fluorescence (Figure 4A). Quantitative analysis showed that the relative intracellular ROS levels in porcine oocytes were significantly higher in the TBTO exposure group than in the control group (Figure 4B; *p* < 0.05).

### TBTO Exposure During the IVM Period Impairs Mitochondrial Function in Porcine Oocytes

**FIGURE 5:**
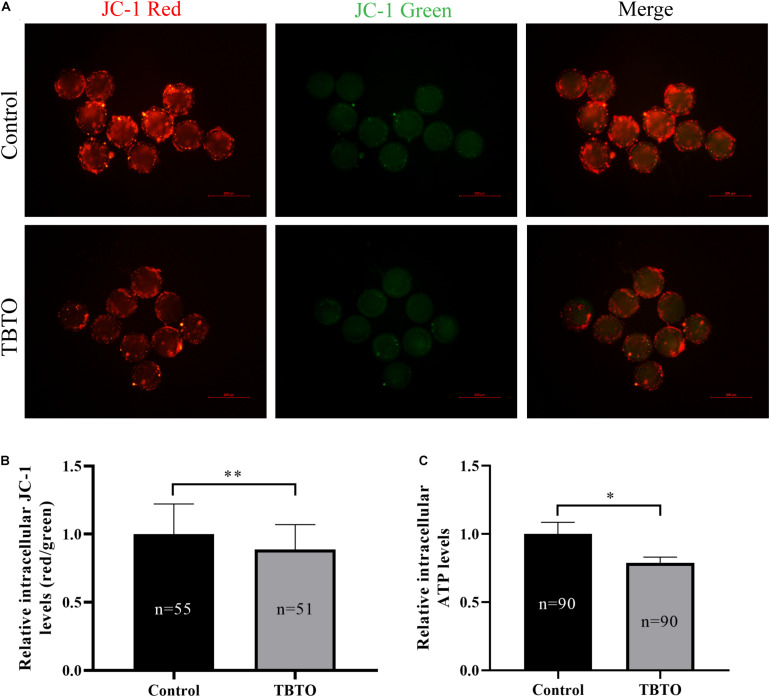
Effects of TBTO exposure on mitochondrial function in porcine oocytes. **(A)** Representative fluorescence images of JC-1-stained porcine oocytes in the control and TBTO exposure group at the end of the IVM period. Scale bar = 200 μm. **(B)** Quantification of the relative JC-1 fluorescence intensity in porcine oocytes from the control and TBTO exposure group. **(C)** Quantification of relative intracellular ATP levels in porcine oocytes from the control and TBTO exposure group. The numbers of oocytes examined from the different groups are indicated by the bars. ^∗^*p* < 0.05; ^∗∗^*p* < 0.01 compared to the control.

Mitochondrial function plays a vital role in oocyte maturation. Therefore, the MitoMP and intracellular ATP levels in porcine oocytes were analyzed. The intracellular MitoMP of porcine oocytes was evaluated using the fluorescent dye JC-1 (Figure 5A). Quantitative analysis showed that there was a loss of MitoMP in porcine oocytes in the TBTO exposure group compared with the control group (Figure 5B; *p < 0.05*). Further analysis showed that the relative intracellular ATP levels in porcine oocytes were significantly lower in the TBTO exposure group than in the control group (Figure 5C; p < 0.05).

### TBTO Exposure During the IVM Period Disrupts Iron Homeostasis in Porcine Oocytes

**FIGURE 6:**
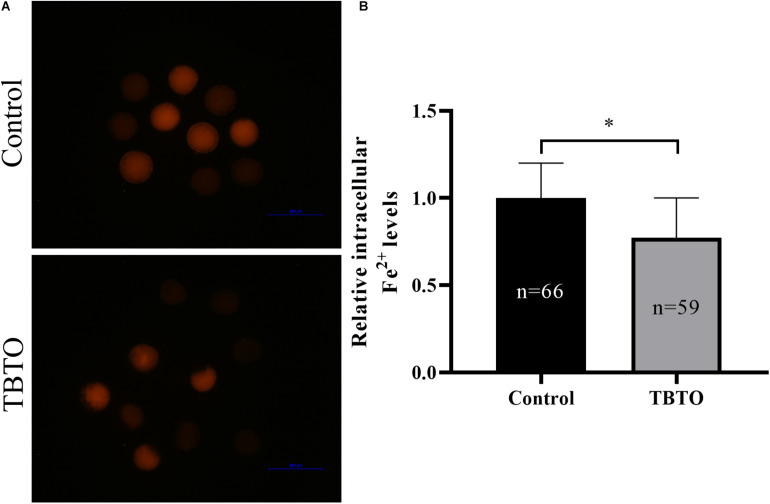
Effect of TBTO exposure on iron homeostasis in porcine oocytes. **(A)** Representative fluorescence images showing intracellular ferrous ion levels in porcine oocytes in the control and TBTO exposure group at the end of the IVM period. **(B)** Quantification of relative intracellular ferrous ion levels in porcine oocytes from the control and TBTO exposure group. The numbers of oocytes examined from the different groups are indicated by the bars. ^∗^*p* < 0.05 compared to the control.

Intracellular ferrous ion levels were measured by using the fluorescence probe Ferro Orange (Figure 6A). Quantitative analysis showed that the relative intracellular ferrous ion levels in porcine oocytes were significantly lower in the TBTO exposure group than in the control group (Figure 6B; *p* < 0.05).

## Discussion

The organotin compound TBTO has been widely used as a biocide in agriculture and as an antifouling agent in paints. In recent years, TBTO has received widespread attention because it is toxic to many cell types. In the present research, we investigated the toxic effects of TBTO exposure on mammalian oocyte quality with a pig model. Our results indicated that TBTO exposure affected porcine oocyte maturation, resulting in excessive accumulation of intracellular ROS, mitochondrial dysfunction, and iron homeostasis disruption in porcine oocytes.

Oocyte maturation is a complex process, and any errors that occur during this process can reduce oocyte quality and preimplantation embryo development potential. The production of high-quality oocytes is vital for effective animal reproduction. Several studies have shown that harmful internal and external factors can decrease oocyte quality and in turn reduce embryonic developmental competence (Liang et al., 2017b; Nie et al., 2019; Sun et al., 2020). The negative effects of TBTO exposure on different types of cells in different species, especially germ cells, have also been reported (Baken et al., 2007; Mochida et al., 2007; Osman and van Loveren, 2012, 2014). Previous research has suggested that TBTO exposure impairs mouse oocyte quality by inducing mitochondrial dysfunction, oxidative stress and apoptosis, thus causing abnormal spindle organization and chromosome alignment and eventually resulting in fertility failure (Yang et al., 2019). In the present study, we used porcine oocytes as a model to investigate the negative effects of TBTO exposure on oocyte quality *in vitro*. The results showed that TBTO exposure affected porcine oocyte maturation, decreasing the proportion of oocytes with PBE by the end of IVM. Further analysis demonstrated that the subsequent developmental potential of porcine oocytes was affected by TBTO exposure. Embryo quality and the proportion of embryos that reached the blastocyst stage were markedly decreased by TBTO exposure during the IVM period after PA. These results support our hypothesis that TBTO exposure during IVM impairs the quality of mature porcine oocytes, thus decreasing the subsequent embryonic developmental competence of the oocytes.

Cumulus cells surrounding oocytes and cumulus expansion play an important role during oocyte meiotic maturation (Chen et al., 1993; Van Soom et al., 2002). During cumulus expansion, cumulus cells synthesize large amounts of hyaluronan, an essential component of the microenvironment necessary for oocyte fertilization (Mlynarcíková et al., 2009). Our results showed that TBTO exposure blocked cumulus expansion in a dose-dependent manner during porcine oocyte maturation. Furthermore, TBTO exposure during IVM dramatically decreased the levels of cumulus expansion-related factors in cumulus cells (COX2 and HAS2). A previous study showed that TBTO exposure upregulates proapoptotic genes and downregulates antiapoptotic genes in mouse thymocytes (Katika et al., 2011). In addition, previous research investigating the relationship between oocyte quality and cumulus cell apoptosis has shown that apoptosis occurring in cumulus cells may be a predictor of oocyte quality and the outcome of *in vitro* fertilization-derived embryo transfer (Lee et al., 2001). In the current study, we found that TBTO exposure increased apoptosis in porcine CCs, as determined at the end of IVM. These findings indicate that TBTO exposure during IVM impairs porcine oocyte quality and decreases subsequent embryonic developmental competence in part by affecting cumulus expansion. However, the detailed underlying mechanism remains to be further elucidated.

Mitochondria play an essential role in oocyte maturation (Babayev and Seli, 2015). However, mitochondrial dysfunction leads to declines in oocyte quality and induces embryonic development failure (Babayev and Seli, 2015; Liang et al., 2017b; Nie et al., 2019; Sun et al., 2020). There is increasing evidence that overproduction of intracellular ROS can cause mitochondrial dysfunction, thus inducing oxidative stress in oocytes (Liang et al., 2017b; Lan et al., 2020; Xu et al., 2020). Previous studies have suggested that TBTO exposure can lead to repression of mitochondrial function in rat thymocytes (Baken et al., 2007) and rat exocrine pancreas tissues (Hara et al., 1994). Porcine oocytes have relatively high intracellular lipid levels, which makes them highly sensitive to oxidative stress-induced impairments (Gajda, 2009). Excessive intracellular ROS accumulation can induce oocyte meiotic arrest, decrease oocyte quality, and reduce subsequent embryonic developmental competence (Tripathi et al., 2009; Zhou et al., 2019). Recent research has suggested that TBTO exposure increases intracellular ROS levels, disrupts mitochondrial distribution, and affects the relative expression of certain genes in mouse oocytes (Yang et al., 2019). In addition, TBTO exposure has been found to activate the oxidative stress response proteins NRF2 and KEAP1 in a human T lymphocyte cell line (Katika et al., 2011). MitoMP is commonly used as an indicator of mitochondrial function in oocytes (Liang et al., 2018) and is the driving force behind intracellular ATP synthesis (Dimroth et al., 2000). Several studies have suggested that an elevated MitoMP in oocytes could be associated with an enhanced oxidative stress response and improved developmental potential (Liang et al., 2017a; An et al., 2019; Nie et al., 2020; Niu et al., 2020). Therefore, we analyzed intracellular ROS accumulation, MitoMP levels and ATP generation in porcine oocytes after TBTO exposure. Our results showed that TBTO exposure not only induced obvious accumulation of intracellular ROS but also caused a loss of MitoMPs and reduced ATP generation in oocytes. These changes may account for the decline in porcine oocyte quality and the reduction in oocyte developmental competence after TBTO exposure.

Iron homeostasis is important for many types of biological processes, such as DNA synthesis by ribonucleotide reductase and cellular respiration by proteins of the mitochondrial electron transport chain (Galaris et al., 2019). Maintaining iron homeostasis is vital for cell proliferation (Weber et al., 2020). Mitochondria play an important role in iron metabolism because they are the intracellular sites of iron–sulfur cluster synthesis (Stehling et al., 2013). Previous studies have suggested that increases in intracellular ROS accumulation and decreases in cellular antioxidant capacity can cause mitochondrial dysfunction, which is characterized by loss of MitoMP, an increase in mitochondrial mass, alteration of mitochondrial respiratory complexes, and an increase in mitochondrial DNA fragmentation (Rizwan et al., 2020). Mitochondrial dysfunction is also accompanied by increased intracellular ROS levels (Han et al., 2001). Elevations in intracellular ROS not only induce mitochondrial dysfunction but also disrupt iron homeostasis (Hughes et al., 2020). Free iron can generate highly reactive ROS directly through the Fenton and Haber–Weiss reactions (Kehrer, 2000). ROS are detoxified by many antioxidant enzymes, such as superoxide dismutase (SOD), glutathione peroxidase (GPX), and catalase (Valko et al., 2007). However, an imbalance between intracellular ROS accumulation and detoxification leads to oxidative stress. Under oxidative stress, excessive ROS can damage iron-sulfur cluster-containing enzymes and ferritin (Vernis et al., 2017; Nakamura et al., 2019). In the present research, we found that TBTO exposure decreased intracellular ferrous ion levels in porcine oocytes. These results suggest that TBTO exposure probably reduces the quality and developmental competence of porcine oocytes by also disrupting iron homeostasis. However, the molecular mechanism underlying this effect remains to be further explored.

## Conclusion

**FIGURE 7:**
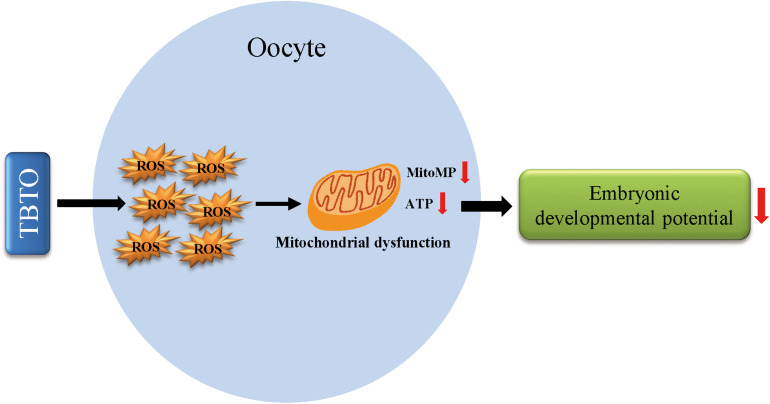
Diagram of the negative effects of TBTO exposure on porcine oocyte maturation. TBTO exposure causes excessive ROS production and induces mitochondrial dysfunction and iron homeostasis disruption, thus impairing oocyte quality and subsequent embryonic developmental competence.

In conclusion, the current study demonstrates that TBTO exposure during porcine oocyte maturation exerts detrimental effects on oocyte quality. Specifically, it causes oxidative stress-induced mitochondrial dysfunction and iron homeostasis disruption in oocytes and eventually leads to a decline in embryonic developmental competence (Figure 7). These findings provide novel insights into the mechanisms underlying TBTO exposure-induced impairments in oocytes. In the future, *in vivo* experiments should be carried out to confirm the toxic effects of TBTO exposure on porcine oocytes and to address the limitations of tests on *in vitro*-matured oocytes.

## Data Availability Statement

The original contributions presented in the study are included in the article/supplementary material, further inquiries can be directed to the corresponding author/s.

## Ethics Statement

The present research followed the Care and Use of Laboratory Animals prepared by the Institutional Animal Care and Use Committee of Jilin University, China.

## Author Contributions

YX, WH, SL, and JZ participated in the research design and wrote the article. YX, WH, JQ, BY, and SL participated in the experiment and data analysis. YX, WH, HJ, BS, JZ, and SL participated in the revised manuscript. All authors contributed to the article and approved the submitted version.

## Conflict of Interest

The authors declare that the research was conducted in the absence of any commercial or financial relationships that could be construed as a potential conflict of interest.
